# Synthesis and Characterization of Privileged Monodentate Phosphoramidite Ligands and Chiral Brønsted Acids Derived from d-Mannitol

**DOI:** 10.3390/ijms13032727

**Published:** 2012-02-29

**Authors:** Abdullah Mohammed A. Al-Majid, Assem Barakat, Yahia Nasser Mabkhot, Mohammad Shahidul Islam

**Affiliations:** Department of Chemistry, Faculty of Science, King Saud University, P.O. Box 2455, Riyadh 11451, Saudi Arabia; E-Mails: yahia@ksu.edu.sa (Y.N.M.); shahid.10amui@gmail.com (M.S.I.)

**Keywords:** phosphoramidite, Brønsted acid, d-mannitol

## Abstract

The synthesis of several novel chiral phosphoramidite ligands (**L1**–**L8**) with *C*_2_ symmetric, pseudo *C*_2_ symmetric secondary amines and chiral Brønsted acids **1a,b** has been achieved. These chiral auxiliaries were obtained from commercially available d-mannitol, and secondary amines in moderate to excellent yields. Excellent diastereoselectivites of ten chiral auxiliaries were obtained. The chiral phosphoramidite ligands and chiral Brønsted acids were fully characterized by spectroscopic methods.

## 1. Introduction

**Figure 1:**
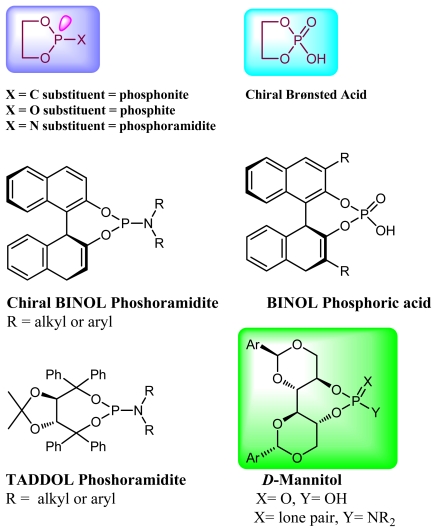
Chiral phosphoramidite ligands and Brønsted acid derived from BINOL or TADDOL backbone.

Asymmetric catalysis is one of the most cost-effective and environmentally friendly methods for the production of a large variety of enantiomerically enriched molecules [1,2]. An important area of research in asymmetric catalysis involves designing enantiopure ligands and transition metal catalysts which can lead to an efficient and selective transformation. Phosphoramidites (Figure 1) have recently attracted considerable interest as ligands in transition-metal catalyzed organic transformations [3–13]. Phosphoramidites are a versatile ligand class, which can serve as two-, four-, six- or eight-electron donors [14]. Privileged monodentate ligands are often based on chiral BINOL or TADDOL backbones (Figure 1), which are combined with phosphorus (III) reagent and a carbon or heteroatom substituent in a modular way [15–24].

The modular assembly makes these ligands suitable for systematic screenings, and that makes general protocols for their rapid synthesis highly desirable. Originally described by Feringa [18], they are increasingly applied as ligands in transition-metal catalyzed organic transformations, such as enantioselective conjugate enone addition reactions [11,25,26], hydrogenations [3,5,6,8], allylic alkylations [9], hydrosilylations [27], vinylations [28], cycloadditions [29–31], Diels-Alder [32] and Heck reactions [33].

We have been developing a new class of chiral monodentate phosphoramidite ligands and chiral Brønsted acid derived from readily accessible enantiopure axially chiral DIOL units (Figure 1). One of the salient features of these novel monodentate phosphorus ligands is their fine-tuning capability through modifications of the R, and Ar groups. This feature is of critical importance because it allows a combinatorial approach to discover the most efficient ligand for a specific reaction or process.

## 2. Results and Discussion

### 2.1. Synthesis of Phosphoramidite Ligands

Our aim was to design and synthesize a library of chiral monophosphoramidite ligands decorated with electron-donating as well as electron-withdrawing groups in addition to sterically-demanding substituents. The general procedure is shown in Table 1. The starting optically-active DIOLs **I** used in these syntheses were prepared according to the literature [34]. The amines used were commercially available or were synthesized from (*R*)-α-methyl benzyl amine according to the literature [35].

**Scheme 1:**
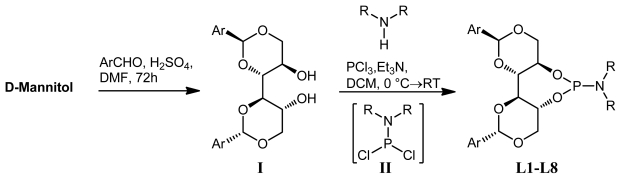
Synthesis of chiral monodentate phosphorus ligands **L1**–**L8**.

The synthetic procedure started with the reaction of amine derivatives with purified PCl_3_ and Et_3_N as base in DCM at 0 °C. The resulting intermediate **II** was treated with one equivalent of DIOLs **I**. The ligands were obtained as white or pale yellow solids or oily products in moderate to good yields (Scheme 1).

The ligands synthesized by this method are shown in Table 1. Ligands **L1** and **L2** were substituted with a diethyl amine group at phosphorus (Table 1, entries 1 and 2). The steric hindrance is even more pronounced in ligand **L2**, with tolyl instead of phenyl moieties in the DIOL **I** backbone. This might also account for the rather low chemical yield (35% as compared to 55%). The ^1^H, ^13^C and ^31^P NMR spectra were as expected for these ligands.

Encouraged by these preliminary results, Ligands **L3**–**L8** were efficiently synthesized in one step using the same methodology related Ligands **L1** and **L2**.

**Figure 2:**
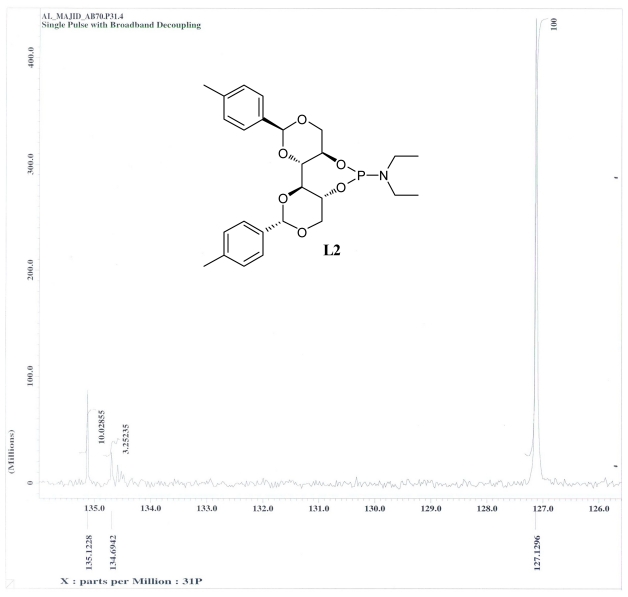
^31^P NMR data of the mixture isomers of **L2**.

The ^31^P NMR spectroscopic data for ligands **L1**–**L8** are summarized in Table 1. It was found that all phosphoramidite ligands were obtained in excellent isomer purity based on ^31^P NMR. In some cases, it was observed that minor product isomers of phosphoramidites are evidenced by ^31^P NMR. Unfortunately, the resulting product oxidized either from aerobic oxidation of the desired phosphoramidite ligands during isolation, or from oxidation of the intermediate dialkylaminophosphorous dichloride (Figure 2). The major and minor isomers of phosphoramidite ligands were not separable by column chromatography. Subsequently, for structure confirmation, the mass spectrum of the new product was recorded. X-ray crystal structure analysis is one possibility to determine the structure unambiguously. Several attempts were made to obtain suitable crystal for X-Ray measurements, but were unsuccessful due to the microcrystalline nature of the products.

Ligand **L1** was obtained by a similar procedure with diethyl amine, using the DCM as the reaction solvent. Similarly, there are four isomers in the mixture, with one isomer dominating the others. The ^31^P NMR analysis identified the major isomer at *δ* = 127.2, while the minor isomers showed chemical shift of *δ* 134.6, 135.12 respectively.

Given that other **L3**, **L4**-phosphoramidites were synthesized, a similar strategy was used with piperidine as secondary amine in 45 and 40% yields respectively. Introduction of *C*_2_ symmetric and pseudo *C*_2_ symmetric secondary amines of the DIOLs **I** scaffold would accomplish the same aims as set out. The phosphoramidites ligands **L1**–**L8** are colorless liquids or white solids, which are readily soluble in common organic solvents (Scheme 1). They were fully characterized by ^1^H, ^13^C and ^31^P NMR spectroscopy, mass spectrometry as well as by elemental analysis. Compounds **L1**–**L8** and their solutions must be kept under anhydrous conditions due to their sensitivity to moisture.

### 2.2. Synthesis of Chiral Brønsted Acids

**Figure 3:**
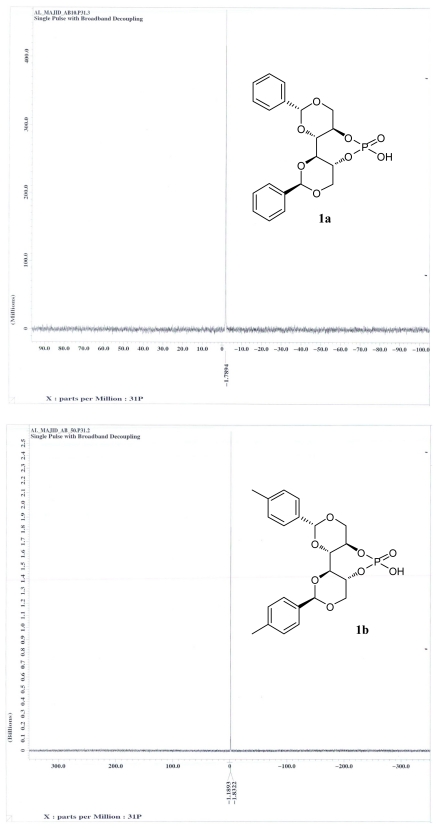
^31^P NMR data of the **1a,b**.

**Scheme 2:**
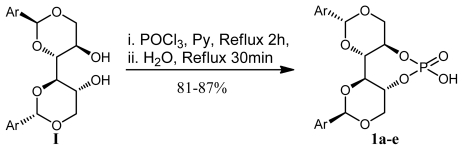
Synthesis of chiral brønsted acids **1a,e**.

Chiral Brønsted acids have emerged as efficient enantioselective catalysts for a variety of organic transformations [35–39]. A critical factor in achieving high stereoselectivities in these transformations is the hydrogen bond formed between the donor site of the acid catalyst and the acceptor (basic) site of the electrophilic component, X-H…Y (X and Y are heteroatoms) [40–45]. In this regard, C-H…X (X = O or N) hydrogen bonding interactions have recently been identified as an important factor in some stereoselective transformations [46–49]. Thus, we decided to synthesize **1a**–**e** and evaluate their utility as a recyclable asymmetric organocatalyst (Scheme 2). Thus, the synthesis of chiral Brønsted acids **1a**–**e** was achieved from DIOL **I** according to procedures set out in the literature [50]. Subsequent reaction of **1a** with POCl_3_ in pyridine at 90 °C, followed by treatment with water and acidification, afforded phosphoric acid **1a** in an excellent overall yield (87%). It should be noted that this reaction is very sensitive to both the concentration of acid, and the time as well. Subsequently, for structure confirmation, a melting point 255 °C for phosphoric acid derivatives **1a** was observed: the temperature for DIOL **I** (entry 1, Table 2, Ph) being 192 °C. The resulting chiral phosphoric acid **1a** was fully characterized by ^1^H, ^13^C, and ^31^P NMR spectroscopy, mass spectrometry as well as by elemental analysis. The ^31^P NMR analysis revealed that only one major product at *δ* = −1.78 was obtained as depicted in Figure 3.

Having identified the optimal reaction conditions, we next examined the scope and limitations of this reaction using various protecting benzylidine moieties with different substituents on the benzene rings; the results are summarized in Table 2. As is shown in Table 2, in the case of the electron-withdrawing group at the 4-position of the benzene ring of DIOL **I**, the reactions proceeded smoothly to give an excellent yield of **1b** (up to 87%) along with excellent diastereoselectivites (Table 2, entry 2). In the case of electron donating group at 4- or at 2,4-positions of the benzene ring of DIOL **I**, no products were obtained (Table 2, entries 3 and 4).

**Scheme 3:**
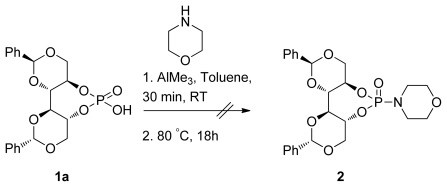
*N*-Morpholino phosphoramidate as a new motif for asymmetric Brønsted acid catalysis.

We are interested in exploring derivatives with alternative acidic and basic sites to further expand the utility of this fascinating type of organocatalyst [51]. Interestingly, when chiral of Brønsted acid **1a** was used to prepare *N*-morpholino phosphoramidate **2**, the reaction failed (Scheme 3).

### 2.3. Applications

**Scheme 4:**
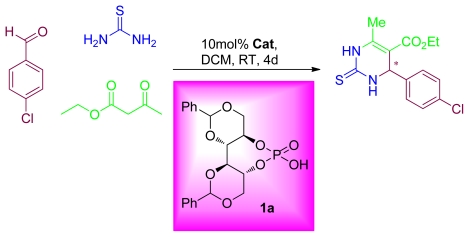
Biginelli reaction.

Chiral dihydropyrimidinethiones (DHPMs) have found increasing applications in the synthesis of pharmaceutically-relevant substances exhibiting a wide range of important pharmacological properties. The Biginelli reaction, one of the most useful multicomponent reactions, offers an efficient way to access multi functionalized 3,4-dihydropyrimidin-2-(1*H*)-ones (DHPMs). Initial screening experiments were performed by applying a Biginelli reaction initiated with the condensation of an aldehyde with urea or thiourea in the presence of a Brønsted acid (Scheme 4). Utilizing 1 equiv. of 4-chlorobenzaldehyde, 1.2 equiv. of thiourea, 3.0 equiv. of ethyl 3-oxobutanoate, and 10 mol% of **1a** in DCM and stirred at RT for 4 days. Formation of dihydropyrimidinethiones (DHPMs) was not observed. Although the reaction was carried out at elevated temperature at 70 °C for 6 days, no catalytic activity was observed. From these initial attempts, it is clear that there is no sign of catalytic activity of **1a** towards Biginelli reaction.

## 3. Experimental Section

**General**: All the moisture and air sensitive reactions were carried out under an inert atmosphere of an argon-filled glove box and standard Schlenk-line techniques. All the chemicals were purchased from Aldrich, Sigma-Aldrich, Fluka *etc.*, and were used without further purification, unless otherwise stated. Toluene was distilled using Na/benzophenone. CH_2_Cl_2_ was dried from CaH_2_. Silica gel (SiO_2_; 100–200 mesh) was used for Flash column chromatography. All melting points were measured on a Gallenkamp melting point apparatus in open glass capillaries and are uncorrected. IR Spectra were measured as KBr pellets on a Nicolet 6700 FT-IR spectrophotometer. The NMR spectra were recorded on a Jeol-400 NMR spectrometer. ^1^H NMR (400 MHz), ^13^C NMR (100 MHz) and ^31^P NMR were run in deuterated dimethylsulphoxide (DMSO-d_6_ or CDCl_3_). Chemical shifts (*δ*) are referred in terms of ppm and *J*-coupling constants are given in Hz. Mass spectra were recorded on a Jeol of JMS-600 H. Elemental analysis was carried out on a Perkin Elmer 2400 Elemental Analyzer; CHN mode. Optical rotations were measured on a Polarimeter, polax-2L.

### 3.1. General Procedure for the Synthesis of *C*_2_ Symmetric and Pseudo *C*_2_ Symmetric Secondary Amines (Procedure A) [35]

A mixture of the appropriately substituted ketone (10 mmol, 1.0 eq.) and amine derivatives (10 mmol, 1.0 eq.) in Ti(Oi-Pr)_4_ (30 mmol, 3.0 eq.) was stirred for 45 min. Pd/C (10%, 200 mg) was added and the mixture stirred under an atmosphere of hydrogen for 48 h. An aqueous solution of NaOH (1 M, 20 mL) was added and the mixture stirred for 45 min. Water (50 mL) was added and the mixture extracted with ethyl acetate (5 × 50 mL). The organic extracts were dried over MgSO_4_, filtered and concentrated to give the desired amine. If necessary, flash chromatography on silica gel (diethyl ether in petroleum ether) could be used to separate diastereomers, though little, if any separation was observed by thin-layer chromatography so, GC analysis is necessary.

### 3.2. (*R*)-Bis((*R*)-1-Phenylethyl) Amine

Following **Procedure A**, (*R*)-bis((*R*)-1-phenylethyl) amine was obtained from acetophenone (1.20 gm, 10 mmol, 1.0 eq.) and (*R*)-*α*-methyl benzyl amine (1.21 gm, 10 mmol, 1.0 eq.) in Ti(O*i*-Pr)_4_ (9.0 mL, 30 mmol, 3.0 eq.) which was obtained as yellowish oil in quantitative yield.

^1^H NMR (400 MHz, CDCl_3_, 21 °C): *δ* = 7.35–7.21 (m, 5 H, C_6_H_5_), 3.51 (q, *J* = 6.6 Hz, 1H, C*H*CH_3_), 2.2 (br, 1H, N*H*), 1.29 (d, *J* = 6.6 Hz, 3H, CHC*H*_3_).

The other analytical data are in accordance with the literature [35].

### 3.3. (*R*)-1-(Naphthalen-2-yl)-*N*-((*R*)-1-Phenylethyl) Ethanamine

Following **Procedure A**, (*R*)-1-(Naphthalen-2-yl)-*N*-((*R*)-1-phenylethyl)ethanamine was obtained from 2-acetonaphthone (1.70 gm, 10 mmol, 1.0 eq.) and (*R*)-α-methyl benzyl amine (1.21 gm, 10 mmol, 1.0 eq.) in Ti(O*i*-Pr)_4_ (9.0 mL, 30 mmol, 3.0 eq.) which was obtained as yellowish oil in quantitative yield.

^1^H NMR (400 MHz, CDCl_3_, 21 °C): *δ* = 7.88 (t, *J* = 9.1 Hz, 2H), 7.76 (d, *J* = 8.1 Hz, 1H), 7.69 (d, *J* = 6.9 Hz, 1H), 7.54–7.23 (m, 6H), 7.18–7.14 (m, 2H), 4.39 (q, *J* = 6.6 Hz, 1H), 3.59 (q, *J* = 6.6 Hz, 1H), 1.37 (d, *J* = 6.6 Hz, 3H), 1.34 (d, *J* = 6.9 Hz, 3H).

The other analytical data are in accordance with the literature [50].

### 3.4. General Procedure for the Preparation of Phosphoramidites (Procedure B)

Triethylamine (7 mmol, 5.0 eq.) was added dropwise to a solution of phosphorus trichloride (1.4 mmol, 1.0 eq.) in dichloromethane (5 mL) at 0 °C. The solution was warmed to room temperature and the amine (1.4 mmol, 1.0 eq.) was added neat as either the free base or HCl salt. The mixture was stirred for 5 h, at which time DIOL **I** (1.4 mmol, 1.0 eq.) was added neat and the mixture stirred overnight. The suspension was concentrated and the ligand purified by flash chromatography on silica gel (dichloromethane in petroleum ether with 1% triethylamine) to give the ligand as an oily substance which solidifies on standing or as a foaming solid.

### 3.5. (4a*R*,7a*R*,11a*S*,11b*S*)-*N,N*-Diethyl-2,10-Diphenylhexahydrobis([1,3]Dioxino)[5,4-d:4′,5′- f][1,3,2]Dioxaphosphepin-6-amine (L1)

Following **Procedure B**, **L1** was obtained from Triethylamine (971 μL, 7 mmol, 5.0 eq.), phosphorus trichloride (123 μL, 1.4 mmol, 1.0 eq.), diethyl amine (102 mg, 143 μL, 1.4 mmol, 1.0 eq.), and (2*S*,2′*S*,4*R*,4′*R*,5*R*,5′*R*)-2,2′-diphenyl-[4,4′-bi(1,3-dioxane)]-5,5′-diol (500 mg, 1.4 mmol, 1.0 eq.) which was obtained as an oily product (355 mg, 0.77 mol, 55%); IR (KBr, cm^−1^): *ν*_max_ = 3436, 1612, 1369; ^1^H NMR (400 MHz, CDCl_3_): *δ* = 7.49–7.34 (m, 5H, Ph), 5.54 (s, 1H, PhC*H*), 4.36 (q, 1H, OC*H*_2_), 4.24 (m, 1H, C*H*O), 3.94 (d, 1H, *J* = 8.8 Hz, OC*H*_2_), 3.81 (m, 1H, C*H*OP), 3.18 (m, 2H, C*H*_2_CH_3_), 1.10 (t, 3H, *J* = 7.3 Hz, C*H*_3_); ^13^C NMR (100 MHz, CDCl_3_): *δ* = 137.3, 128.3, 126.2, 126.1, 100.7, 100.4, 82.8, 81.6, 38.6, 38.4; ^31^P NMR (130 MHz, CDCl_3_): *δ* = 127.2; MS (*m/z)*: 460.47 [M + 1]_+_, 47%; Anal. for C_24_H_30_NO_6_P; calcd: C, 62.74; H, 6.58; N, 3.05. Found: C, 62.50; H, 6.49; N, 3.00.

### 3.6. (4a*R*,7a*R*,11a*S*,11b*S*)-*N,N*-Diethyl-2,10-di-*p*-Tolylhexahydrobis([1,3]Dioxino)[5,4-d:4′,5′- f][1,3,2]Dioxaphosphepin-6-amine (L2)

Following **Procedure B**, **L2** was obtained from Triethylamine (971 μL, 7 mmol, 5.0 eq.), phosphorus trichloride (123 μL, 1.4 mmol, 1.0 eq.), diethyl amine (102 mg, 143 μL, 1.4 mmol, 1.0 eq.), and (2*S*,2′*S*,4*R*,4′*R*,5*R*,5′*R*)-2,2′-di-*p*-tolyl-[4,4*′*-bi(1,3-dioxane)]-5,5*′*-diol (541 mg, 1.4 mmol, 1.0 eq.) which was obtained as a foaming white solid (265 mg, 0.49 mol, 35%); m.p.: 65 °C; IR (KBr, cm^−1^): *ν*_max_ = 3435, 1610, 1345; ^1^H NMR (400 MHz, CDCl_3_): *δ* = 7.37–7.34 (m, 2H, Ph), 7.17–7.14 (m, 2H, Ph), 5.46 (s, 1H, PhC*H*), 4.33(q, 1H, OC*H*_2_), 4.22 (m, 1H, C*H*O), 3.89 (d, 1H, *J* = 8.8 Hz, OC*H*_2_), 3.76 (m, 1H, C*H*OP), 3.21–3.16 (m, 2H, C*H*_2_CH_3_), 2.36 (s, 3H, C_6_H_4_C*H*_3_), 1.09 (t, 3H, *J* = 6.6 Hz, C*H*_3_); ^13^C NMR (100 MHz, CDCl_3_): *δ* = 138.7, 134.6, 128.9, 126.1, 100.7, 100.5, 82.8, 81.5, 38.6, 21.3, 14.8; ^31^P NMR (130 MHz, CDCl_3_): *δ* = 127.1; MS (*m/z*): 488.55 [M + 1]_+_, 40%; Anal. for C_26_H_34_NO_6_P; calcd: C, 64.05; H, 7.03; N, 2.87. Found: C, 64.00; H, 7.00; N, 2.95.

### 3.7. 1-((4a*R*,7a*R*,11a*S*,11b*S*)-2,10-Diphenylhexahydrobis([1,3]dioxino)[5,4-d:4′,5′- f][1,3,2]dioxaphosphepin-6-yl)piperidine (L3)

Following Procedure B, **L3** was obtained from Triethylamine (971 μL, 7 mmol, 5.0 eq.), phosphorus trichloride (123 μL, 1.4 mmol, 1.0 eq.), piperidine (121 mg, 1.4 mmol, 1.0 eq.), and (2*S*,2′*S*,4*R*,4′*R*,5*R*,5′*R*)-2,2′-diphenyl-[4,4′-bi(1,3-dioxane)]-5,5′-diol (500 mg, 1.4 mmol, 1.0 eq.) which was obtained as a foaming white solid (265 mg, 0.49 mol, 35%); m.p.: 110 °C; IR (KBr, cm^−1^): *ν*_max_ = 3444, 1607, 1350; ^1^H NMR (400 MHz, CDCl_3_): *δ* = 7.53–7.31 (m, 5H, Ph), 5.50 (s, 1H, PhC*H*), 4.37 (q, 1H, OC*H*_2_), 4.24 (m, 1H, C*H*O), 3.91 (d, 1H, *J* = 8.8 Hz, OC*H*_2_), 3.79 (m, 1H, C*H*OP), 3.19 (m, 2H, C*H*_2_CH_2_), 1.63 (m, 2H, CH_2_C*H*_2_CH_2_), 1.49 (m, 2H, CH_2_CH_2_C*H*_2_); ^13^C NMR (100 MHz, CDCl_3_): *δ* = 137.7, 129.0, 128.3, 126.2, 100.9, 82.8, 82.1, 76.7, 45.6, 27.2, 25.2; ^31^P NMR (130 MHz, CDCl_3_): *δ* = 122.86; MS (*m/z*): 472.18 [M + 1]^+^, 30%; Anal. for C_25_H_30_NO_6_P; calcd: C, 63.69; H, 6.41; N, 2.97. Found: C, 63.55; H, 6.35; N, 2.90.

### 3.8. 1-((4a*R*,7a*R*,11a*S*,11b*S*)-2,10-Di-*p*-Tolylhexahydrobis([1,3]dioxino)[5,4-d:4′,5′- f][1,3,2]dioxaphosphepin-6-yl)piperidine (L4)

Following Procedure B, **L4** was obtained from Triethylamine (971 μL, 7 mmol, 5.0 eq.), phosphorus trichloride (123 μL, 1.4 mmol, 1.0 eq.), piperidine (121 mg, 1.4 mmol, 1.0 eq.), and (2*S*,2′*S*,4*R*,4′*R*,5*R*,5′*R*)-2,2′-di-p-tolyl-[4,4′-bi(1,3-dioxane)]-5,5′-diol (541 mg, 1.4 mmol, 1.0 eq.) which was obtained as a foaming white solid (150 mg, 0.30 mol, 40%); m.p.: 100 °C; IR (KBr, cm^−1^): *ν*_max_ = 3443, 1600, 1339; ^1^H NMR (400 MHz, CDCl_3_): *δ* = 7.38–7.33 (dd, 2H, Ph), 7.18–7.16 (dd, 2H, Ph), 5.45 (s, 1H, PhC*H*), 4.37(q, 1H, OC*H*_2_), 4.24 (m, 1H, C*H*O), 3.80 (d, 1H, *J* = 8.8 Hz, OC*H*_2_), 3.73 (m, 1H, C*H*OP), 3.19 (m, 2H, C*H*_2_CH_2_), 2.32 (s, 3H, CH_3_), 1.63 (m, 2H, CH_2_C*H*_2_CH_2_), 1.49 (m, 2H, CH_2_CH_2_C*H*_2_); ^13^C NMR (100 MHz, CDCl_3_): *δ* = 138.9, 133.3, 128.9, 126.1, 100.9, 82.8, 81.5, 77.4, 28.6, 21.3; ^31^P NMR (130 MHz, CDCl_3_): *δ* = 122.86; MS (*m/z*): 500.21 [M + 1]^+^, 75%; Anal. for C_27_H_34_NO_6_P; calcd: C, 64.92; H, 6.86; N, 2.80. Found: C, 65.02; H, 6.75; N, 2.65.

### 3.9. (4a*R*,7a*R*,11a*S*,11b*S*)-2,10-Diphenyl-*N,N*-bis((*S*)-1-phenylethyl)hexahydrobis([1,3]dioxino) [5,4-d:4′,5′-f][1,3,2]dioxaphosphepin-6-amine (L5)

Following Procedure B, **L5** was obtained from Triethylamine (971 μL, 7.0 mmol, 5.0 eq.), phosphorus trichloride (123 μL, 1.4 mmol, 1.0 eq.), (*R*)-bis((*R*)-1-phenylethyl) amine (315 mg, 1.4 mmol, 1.0 eq.), and (2*S*,2′*S*,4*R*,4′*R*,5*R*,5′*R*)-2,2′-diphenyl-[4,4′-bi(1,3-dioxane)]-5,5′-diol (500 mg, 1.4 mmol, 1.0 eq.) which was obtained as a foaming white solid (200 mg, 0.44 mmol, 31%); m.p.: 103 °C; IR (KBr, cm^−1^): *ν*_max_ = 3423, 1625, 1310; ^1^H NMR (400 MHz, CDCl_3_): *δ* = 7.53–7.34 (m, 10H, Ph), 5.50 (s, 1H, PhC*H*), 4.65 (m, 1H, C*H*CH_3_), 4.25(q, 1H, OC*H*_2_), 3.97 (m, 1H, C*H*O), 3.91 (d, 1H, *J* = 8.8 Hz, OC*H*_2_), 3.80 (m, 1H, C*H*OP), 1.21 (d, 3H, *J* = 8.8 Hz, C*H*_3_); ^13^C NMR (100 MHz, CDCl_3_): *δ* = 137.0, 128.3, 126.2, 100.8, 82.5, 80.6, 69.5, 31.0, 29.7; ^31^P NMR (130 MHz, CDCl_3_): *δ* = 134.65; MS (*m/z*): 612.24 [M + 1]^+^, 64%; Anal. for C_36_H_38_NO_6_P; calcd: C, 70.69; H, 6.26; N, 2.29. Found: C, 70.69; H, 6.45; N, 2.33.

### 3.10. (4a*R*,7a*R*,11a*S*,11b*S*)-*N,N*-Bis((*S*)-1-Phenylethyl)-2,10-di-*p*tolylhexahydrobis([ 1,3]dioxino)[5,4-d:4′,5′-f][1,3,2]dioxaphosphepin-6-amine (L6)

Following Procedure B, **L6** was obtained from Triethylamine (971 μL, 7.0 mmol, 5.0 eq.), phosphorus trichloride (123 μL, 1.4 mmol, 1.0 eq.), (*R*)-bis((*R*)-1-phenylethyl) amine (315 mg, 1.4 mmol, 1.0 eq.), and (2*S*,2′*S*,4*R*,4′*R*,5*R*,5′*R*)-2,2′-di-p-tolyl-[4,4′-bi(1,3-dioxane)]-5,5′-diol (541 mg, 1.4 mmol, 1.0 eq.) which was obtained as a foaming white solid (400 mg, 0.62 mmol, 45%); m.p.: 80–82 °C; IR (KBr, cm^−1^): *ν*_max_ = 3441, 1618, 1343; ^1^H NMR (400 MHz, CDCl_3_): *δ* = 7.43–7.04 (m, 9H, Ph), 5.52 (s, 1H, PhC*H*), 4.61 (m, 1H, C*H*O), 4.42(q, 1H, OC*H*_2_), 4.25(q, 1H, OC*H*_2_), 4.04 (m, 1H, C*H*CH_3_), 3.80 (m, 1H, C*H*OP), 2.33(s, 3H, C*H*_3_), 1.21 (d, 3H, *J* = 8.8 Hz, C*H*_3_); ^13^C NMR (100 MHz, CDCl_3_): *δ* = 143.0, 139.5, 134.5, 128.9, 127.9, 127.8, 126.7, 100.7, 82.9, 81.7, 29.7, 21.3; ^31^P NMR (130 MHz, CDCl_3_): *δ* = 132.5; MS (*m/z*): 640.22 [M + 1]^+^, 55%; Anal. for C_38_H_42_NO_6_P; calcd: C, 71.35; H, 6.62; N, 2.19. Found: C, 71.29; H, 6.50; N, 2.13.

### 3.11. (4a*R*,7a*R*,11a*S*,11b*S*)-*N*-((*S*)-1-(Naphthalen-2-yl)ethyl)-2,10-diphenyl-*N*-((*S*)-1- Phenylethyl)hexahydrobis([1,3]dioxino)[5,4-d:4′,5′-f][1,3,2]dioxaphosphepin-6-amine (L7)

Following Procedure B, **L7** was obtained from triethylamine (971 μL, 7.0 mmol, 5.0 eq.), phosphorus trichloride (123 μL, 1.4 mmol, 1.0 eq.), (*R*)-1-(naphthalen-2-yl)-*N*-((*R*)-1-phenylethyl) ethanamine (315 mg, 1.4 mmol, 1.0 eq.), and (2*S*,2′*S*,4*R*,4′*R*,5*R*,5′*R*)-2,2′-diphenyl-[4,4′-bi(1,3-dioxane)]-5,5′-diol (500 mg, 1.4 mmol, 1.0 eq.) which was obtained as a foaming white solid (463 mg, 0.7 mmol, 50%); m.p.: 98 °C; IR (KBr, cm^−1^): *ν_max_* = 3435, 1632, 1299; ^1^H NMR (400 MHz, CDCl_3_): *δ* = 7.88–7.35 (m, 12H, Ph), 5.53 (s, 1H, PhC*H*), 4.57 (m, 1H, C*H*CH_3_), 4.25(q, 1H, OC*H***_2_**), 4.11 (m, 1H, C*H*O), 4.00 (d, 1H, *J* = 8.8 Hz, OC*H*_2_), 3.79 (m, 1H, C*H*OP), 1.31 (d, 3H, *J* = 8.8 Hz, C*H*_3_), 1.21 (d, 3H, *J* = 8.8 Hz, C*H*_3_) ; ^13^C NMR (100 MHz, CDCl_3_): *δ* = 137.5, 129.1, 128.3, 126.2, 126.1, 100.8, 82.5, 80.7, 69.7, 61.8,53.2, 21.3; ^31^P NMR (130 MHz, CDCl_3_): *δ* = 135.01; MS (*m/z*): 662.26 [M + 1]^+^, 35%; Anal. for C_40_H_40_NO_6_P; calcd: C, 72.60; H, 6.09; N, 2.12. Found: C, 72.48; H, 6.00; N, 2.08.

### 3.12. (4a*R*,7a*R*,11a*S*,11b*S*)-*N*-((*S*)-1-(Naphthalen-2-yl)ethyl)-*N*-((*S*)-1-phenylethyl)-2,10-di-*p*tolylhexahydrobis([ 1,3]dioxino)[5,4-d:4′,5′-f][1,3,2]dioxaphosphepin-6-amine (L8)

Following Procedure B, **L8** was obtained from Triethylamine (971 μL, 7.0 mmol, 5.0 eq.), phosphorus trichloride (123 μL, 1.4 mmol, 1.0 eq.), (*R*)-1-(naphthalen-2-yl)-*N*-((*R*)-1-phenylethyl) ethanamine (315 mg, 1.4 mmol, 1.0 eq.), and (2*S*,2′*S*,4*R*,4′*R*,5*R*,5′*R*)-2,2′-di-*p*-tolyl-[4,4′-bi(1,3- dioxane)]-5,5′-diol (541 mg, 1.4 mmol, 1.0 eq.) which was obtained as a foaming white solid (366 mg, 0.53 mmol, 38%); m.p.: 85 °C; IR (KBr, cm^−1^): *ν_max_* = 3436, 1615, 1378; ^1^H NMR (400 MHz, CDCl_3_): *δ* = 7.88–7.16 (m, 12H, Ph), 5.47 (s, 1H, PhC*H*), 4.50 (m, 1H, C*H*CH_3_), 4.39(q, 1H, OC*H***_2_**), 4.24 (m, 1H, C*H*O), 4.10 (d, 1H, *J* = 8.8 Hz, OC*H*_2_), 3.80 (m, 1H, C*H*OP), 2.36 (s, 3H, C*H*_3_), 1.31 (d, 3H, *J* = 8.8 Hz, C*H*_3_), 1.25 (d, 3H, *J* = 8.8 Hz, C*H*_3_); ^13^C NMR (100 MHz, CDCl_3_): *δ* = 137.5, 129.1, 128.3, 126.2, 126.1, 100.8, 82.5, 80.7, 69.7, 61.8,53.2, 21.5, 21.3; ^31^P NMR (130 MHz, CDCl_3_): *δ* = 134.69; MS (*m/z*): 690.29 [M + 1]^+^, 70%; Anal. for C_42_H_44_NO_6_P; calcd: C, 73.13; H, 6.43; N, 2.03. Found: C, 73.40; H, 6.27; N, 2.05.

### 3.13. General Procedure for the Preparation of Chiral Brønsted Acid (Procedure C) [20]

To a solution of DIOL **I** (0.5 g, 1.29 mmol) in dry pyridine (10 mL) was slowly added phosphoryl chloride (178 μL, 1.94 mmol, 1.5 equiv.) at room temperature and the mixture was heated to reflux for 2 h. The reaction mixture was then allowed to cool to room temperature. Distilled water (0.83 mL) was added, and then the mixture was heated to 95 °C for 30 min and cooled again to room temperature. Pyridine was removed *in vacuo*, and 6 M HCl was added to the mixture. The mixture was extracted with CH_2_Cl_2_, and the combined organic extracts were again washed with 6 M HCl solution 3 times, and dried over anhydrous Na_2_SO_4_, and concentrated *in vacuo*. The crude residue was purified by column chromatography on SiO_2_ (hexane:AcOEt = 3:1→CH_2_Cl_2_:MeOH = 4:1, v:v) to give the desired compound.

### 3.14. (4a*R*,7a*R*,11a*S*,11b*S*)-6-Hydroxy-2,10-diphenylhexahydrobis([1,3]dioxino)[5,4-d:4′,5′- f][1,3,2]Dioxaphosphepine 6-Oxide *(1a)*

Following **Procedure C**, **1a** was obtained from (2*S*,2′*S*,4*R*,4′*R*,5*R*,5′*R*)-2,2′-diphenyl-[4,4′-bi(1,3- dioxane)]-5,5′-diol as a white solid (471 mg, 1.12 mmol, 87%); m.p.: 270 °C; IR (KBr, cm^−1^): *ν_max_* = 3450, 1610, 1355, 1200; [α] *_D_*^24^ = +77° (*c* = 1.0 g/dL, DMSO); ^1^H NMR (400 MHz, CDCl_3_): *δ* = 7.41–7.35 (m, 5H, Ph), 5.65 (s, 1H, PhC*H*), 4.60(brs, 1H, O*H*), 4.28(q, 1H, *J* = 11.0 Hz, OC*H***_2_**), 4.17 (m, 1H, OC*H*), 4.04 (d, 1H, *J* = 8.8 Hz, OC*H*_2_), 3.79 (t, 1H, *J* = 10.2 Hz, C*H*OP); ^13^C NMR (100 MHz, CDCl_3_): *δ* = 137.7, 129.5, 128.7, 126.8, 100.4, 80.6, 68.4, 68.3, 65.7; ^31^P NMR (130 MHz, CDCl_3_): *δ* = −1.78; MS (*m/z*): 421.10 [M + 1]^+^, 85%; Anal. for C_20_H_21_O_8_P; calcd: C, 57.15; H, 5.04. Found: C, 57.20; H, 5.00.

### 3.15. (4a*R*,7a*R*,11a*S*,11b*S*)-6-Hydroxy-2,10-di-*p*-tolylhexahydrobis([1,3]dioxino)[5,4-d:4′,5′- f][1,3,2]Dioxaphosphepine 6-Oxide (1b)

Following **Procedure C**, **1b** was obtained from (2*S*,2′*S*,4*R*,4′*R*,5*R*,5′*R*)-2,2′-di-*p*-tolyl-[4,4′-bi(1,3- dioxane)]-5,5′-diol as a white solid (470 mg, 1.04 mmol, 81%); m.p.: 255 °C; IR (KBr, cm^−1^): *ν_max_* = 3451, 1612, 1369, 1210; [α]*_D_*^24^ = +58° (*c =* 1.0 g/dL, DMSO); ^1^H NMR (400 MHz, CDCl_3_): *δ* = 7.27 (d, 2H, *J* = 8.0 Hz, Ph), 7.16 (d, 2H, *J* = 8.0 Hz, Ph), 5.58 (s, 1H, PhC*H*), 4.60 (brs, 1H, O*H*), 4.25 (q, 1H, *J* = 11.0 Hz, OC*H***_2_**), 4.13 (m, 1H, OC*H*), 4.04 (dd, 1H, *J* = 8.8 Hz, OC*H*_2_), 3.79 (t, 1H, *J* = 10.2 Hz, C*H*OP), 2.27 (s, 3H, C*H*_3_); ^13^C NMR (100 MHz, CDCl_3_): *δ* = 138.8, 134.9, 129.1, 126.7, 100.5, 80.6, 68.4, 65.7, 21.3; ^31^P NMR (130 MHz, CDCl_3_): *δ* = −1.83; MS (*m/z*): 449.13 [M + 1]^+^, 76%; Anal. for C_22_H_25_O_8_P; calcd: C, 58.93; H, 5.62. Found: C, 58.73; H, 5.55.

## 4. Conclusions

We have designed chiral phosphoramidites **L1**–**L8** and Brønsted acid **1a,b** as a new motif for asymmetric catalysis. The potentially broad utility of this motif will be further explored in our laboratory.

## Figures and Tables

**Table 1:** Results of synthesis of chiral phosphoramidite ligands.

#	Compound	Ligand	Ar	δ P a	Yield [%] b
1	**L1**	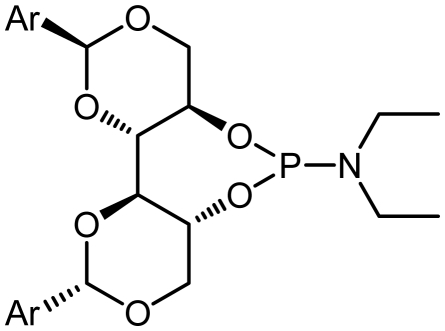	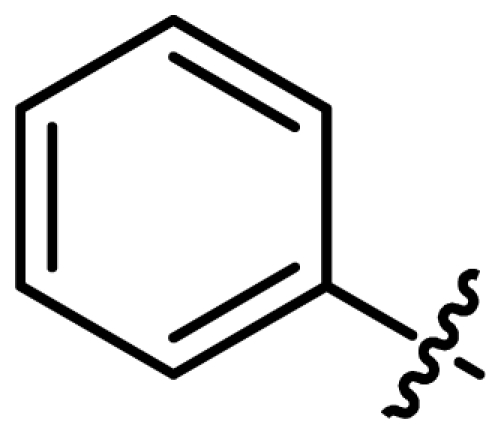	127.2	55
2	**L2**	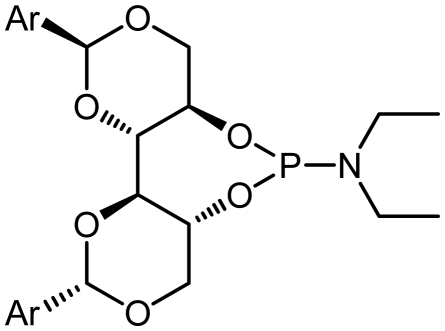	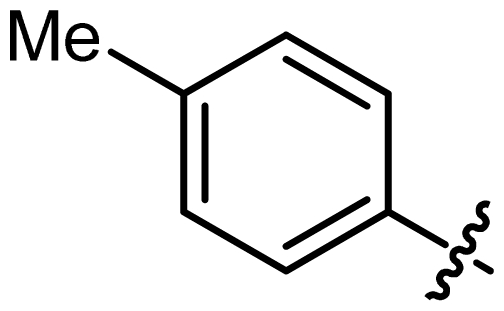	127.12	35
3	**L3**	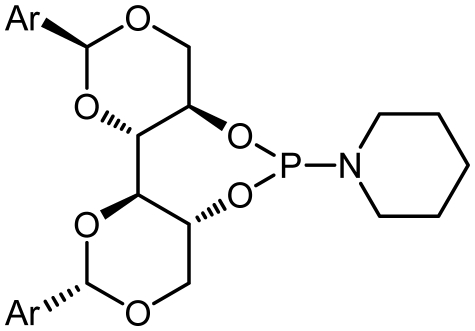	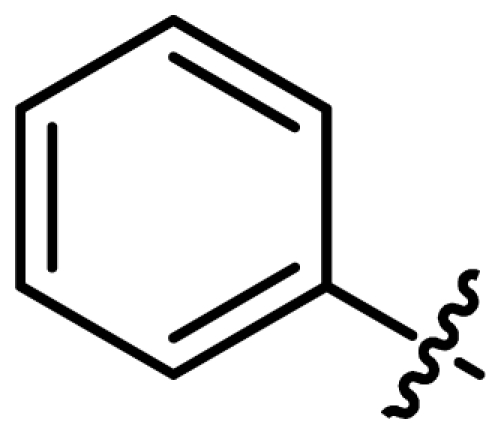	122.86	45
4	**L4**	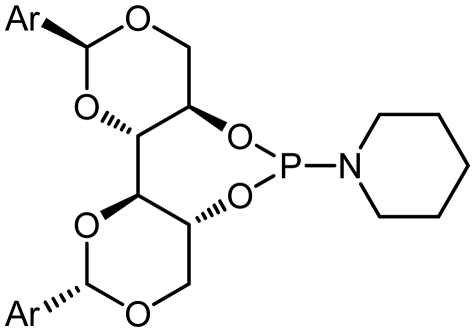	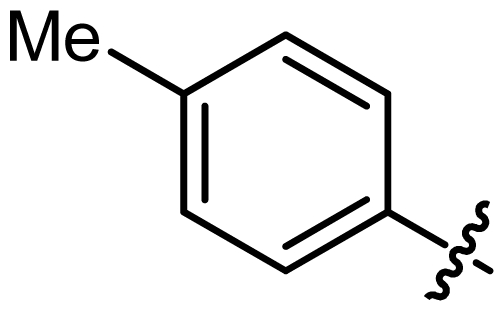	122.60	40
5	**L5**	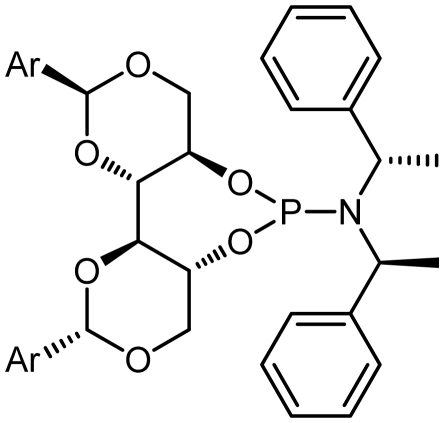	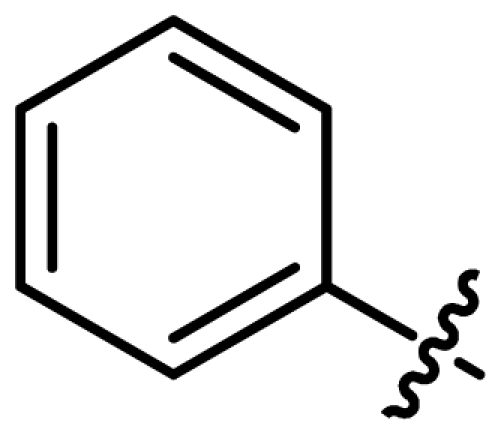	134.65	31
6	**L6**	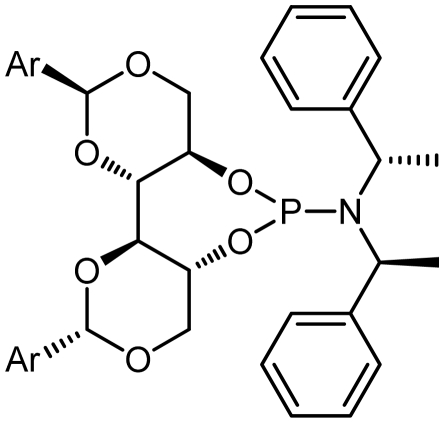	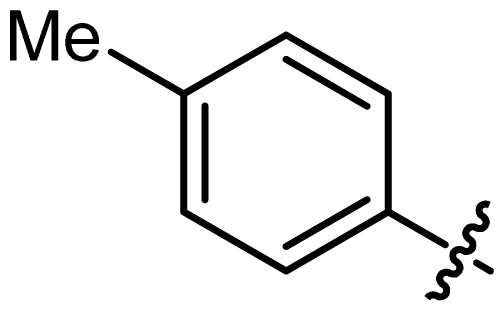	132.50	45
7	**L7**	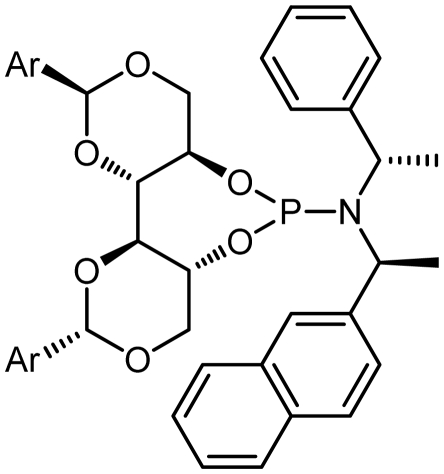	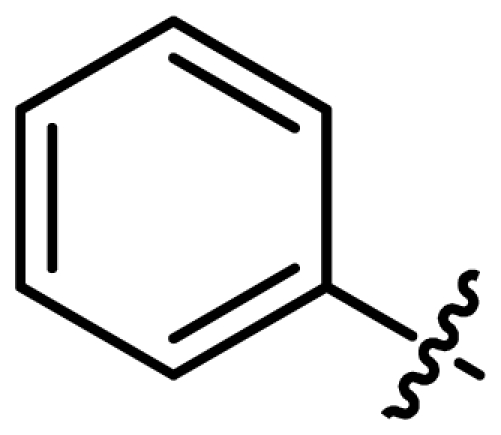	135.01	50
8	**L8**	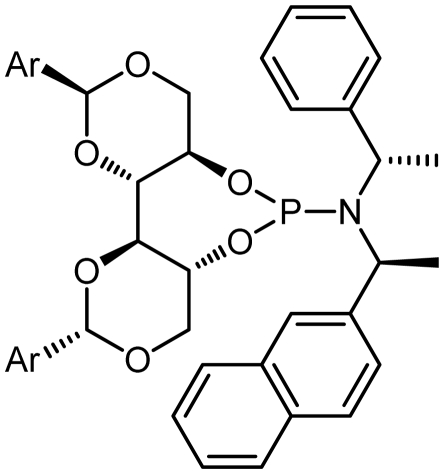	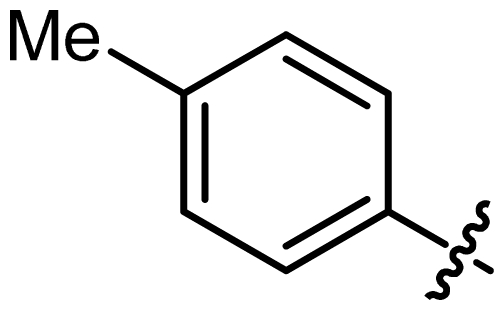	134.69	38

a Determined by ^31^P NMR;

b Isolated yield after column chromatography.

**Table 2:** Results of synthesis of chiral Brønsted acids having aromatic groups in the auxiliary.

#	Compound 1	Ar	δ P a	Yield [%] b
1	**a**	C_6_H_5_	−1.78	87
2	**b**	*p*-CH_3_C_6_H_4_	−1.83	81
3	**c**	*p*-CH_3_OC_6_H_4_	-	-
4	**e**	2,4-diClC_6_H_3_	-	-

a Determined by ^31^P NMR;

b Isolated yield after column chromatography;

-: no product isolated.
